# Toxin Mediated Diarrhea in the 21^st^ Century: The Pathophysiology of Intestinal Ion Transport in the Course of ETEC, *V. cholerae* and Rotavirus Infection

**DOI:** 10.3390/toxins2082132

**Published:** 2010-08-10

**Authors:** Sascha Kopic, John P. Geibel

**Affiliations:** Departments of Surgery & Cellular and Molecular Physiology, Yale University School of Medicine, New Haven, CT, USA; Email: sascha.kopic@yale.edu

**Keywords:** cholera, enterotoxigenic *E. coli*, enterotoxin, NSP4, STa, LT-I

## Abstract

An estimated 4 billion episodes of diarrhea occur each year. As a result, 2–3 million children and 0.5–1 million adults succumb to the consequences of this major healthcare concern. The majority of these deaths can be attributed to toxin mediated diarrhea by infectious agents, such as *E. coli*, *V. cholerae* or Rotavirus. Our understanding of the pathophysiological processes underlying these infectious diseases has notably improved over the last years. This review will focus on the cellular mechanism of action of the most common enterotoxins and the latest specific therapeutic approaches that have been developed to contain their lethal effects.

## 1. Introduction

Diarrhea is widely accepted as a major healthcare concern. However, only closer examination of the epidemiological data available to us fully illustrates the massive burden that acute diarrheal illness (ADI) places on our society. Children are particularly vulnerable to the lethal effects of ADI: 3.2 episodes of diarrhea occur per child/year in the age group <5 years [1]. A total of 2.5 million ADI related deaths worldwide (<5 years) occur each year [1]. This means that one out of five deaths in children (<5 years) is caused by diarrhea and is, in theory, preventable [1]. Although the specific infectious agent cannot always be identified, the majority of ADI cases are attributable to Rotavirus and *E. coli* infections.

In the course of this brief review we will revisit the physiological processes underlying intestinal ion transport and proceed to elucidate how prominent enterotoxigenic bacteria manipulate the cellular biology to induce diarrhea. This review will focus on two bacterial and one viral agent that will serve as a *pars pro toto* for the most common intracellular messaging cascades that are perturbed by enterotoxins: enterotoxigenic *E. coli* (ETEC) and its heat stable enterotoxin (STa) for cGMP induced diarrhea, *V. cholerae* for cAMP induced diarrhea, and Rotavirus for Ca^2+^ induced diarrhea. We will additionally discuss the latest specific pharmacological approaches that have been developed to contain the lethal effects of toxin mediated diarrhea. 

## 2. The Physiology of Intestinal Ion Transport

In order to fully understand the pathophysiological events that occur during toxin mediated diarrhea, one must appreciate the physiology of intestinal ion and water transport. The enterocyte, which is organized in a columnar epithelial monolayer, is responsible for both electrolyte absorption and secretion in the intestine. Although intestinal electrolyte secretion mainly occurs in the crypt and electrolyte absorption takes place in the villus or surface epithelium, some overlap in terms of function is reported to exist between the two regions [2,3,4,5,6]. To account for the conciseness of this overview, the term enterocyte will be used without consideration for functional variability within the mucosa or the intestine in general. 

### 2.1. Enterocyte Electrolyte Absorption

Apical electrolyte absorption by the enterocyte can be divided into two major categories: (i) electroneutral absorption and (ii) electrogenic absorption [5]. 

Electroneutral absorption is a synchronized event that involves Na^+^ uptake by the Na^+^, H^+^-Exchanger (NHE) and Cl^−^ uptake by Cl^−^,HCO_3_^-^-Exchangers. Currently, nine isoforms of NHE are known (for reviews please see [7,8]). NHE1 is located on the basolateral surface of the enterocyte and primarily functions as a housekeeping protein for pH and volume homeostasis, whereas NHE2 and NHE3 have an apical localization pattern and conduce to bulk Na^+^ absorption [5,7,9,10,11,12,13]. Although the relative contribution of NHE2 and NHE3, respectively, varies between both species and localization within the intestine, NHE3 is thought to be the main mediator of electroneutral Na^+^ uptake [7]. This assumption is further underscored by the development of NHE2 and NHE3 knockout mouse models. In NHE2 (−/−) animals intestinal Na^+^ uptake was unaffected. Still, compensatory elevations of NHE3 mRNA and protein levels were reported [14]. Conversely, NHE3 (−/−) animals showed a strong intestinal phenotype with intestinal hypertrophy and diarrhea, thereby emphasizing the significance of NHE3 for physiological salt absorption [14]. Recently, NHE8 has emerged as an important candidate for Na^+^ absorption in immature animals, which may account for the observed residual Na^+^ uptake in NHE2/3 (−/−) animals [15,16]. NHE activity is chiefly regulated by cyclic nucleotides, most notably cAMP. Elevations in cAMP and activation of protein kinase A (PKA) were both shown to inhibit NHE3 [5,7]. For effective inhibition to take place, complex formation with accessory proteins, such as NHERF1/2 and the cytoskeleton linker ezrin, is essential [17,18,19]. A close coupling of NHE activity to chloride transport and in particular the cystic fibrosis transmembrane conductance regulator (CFTR) channel has also been discussed [20,21]. The importance of this interaction was further supported by the ineffectiveness of cAMP in inhibiting Na^+^ uptake in CFTR (−/−) animals. Interestingly, it has been suggested that NHERF1 also interacts with CFTR and is pivotal for NHE inhibition in the pancreas [22]. CFTR, NHERF1 and NHE3 may thus form an inhibitory complex in response to increased levels of cAMP [22]. 

Chloride uptake through apical exchange proteins represents the second component of electroneutral absorption. Two members of the solute carrier 26 (SLC26) gene family are expressed on the apical enterocyte membrane: SLC26A3 (also known as DRA) and SLC26A6 (also known as PAT1)[23]. PAT1 expression is predominant in the small intestine, whereas DRA expression is higher in the colon [24,25]. Both exchangers can transport HCO_3_^−^ in exchange for Cl^−^, but their stoichiometry differs in that PAT1 transports 2 HCO_3_^−^, whereas DRA transports 2 Cl^−^ [26,27]. Despite divergent stoichiometry, the concerted uptake of both exchangers is postulated to be electroneutral. In general, the role of DRA in intestinal absorption is better characterized than PAT1. Mutations of DRA are known to be responsible for autosomal recessive congenital chloride diarrhea (CLD, OMIM 214700)[28,29]. CLD was first described in 1945 in patients exhibiting diarrhea with increased chloride content in the stool. Today, more than 250 cases which are mainly clustered in Finland, Poland and Saudi Arabia, have been reported and 30 mutations in DRA that are responsible for CLD have been identified [29]. Both PAT1 and DRA are regulated by CFTR [27,30]. In transfected cells stimulation of CFTR by cAMP leads to a six-fold increase in activity of both exchangers [27]. Additionally, DRA has been shown to be regulated by intracellular Ca^2+^ [31]. Until recently, some controversy persisted regarding the relative contribution and importance of DRA and PAT1, respectively [24,32]. Functional studies in DRA and PAT1 (−/−) animals have now identified DRA as the predominant mediator of electroneutral chloride uptake in the small intestine [30,33]. Members of the SLC4 gene family represent the second type of enterocytic anion exchange. SLC4A1 (AE1) localization has been demonstrated on the apical surface of colonic enterocytes in the rat [34,35]. These findings could not be reproduced in humans, when investigating mRNA levels throughout the entire intestine [36]. The contribution of AE1 to physiological Cl^−^ uptake thus remains questionable. SLC4A9 (AE4) was also identified in the duodenum, yet subsequent knock-out studies showed that overall HCO_3_^-^ transport was decreased by only <5% in affected animals [25,37].

Electrogenic electrolyte absorption accounts for the second source of apical ion uptake and can occur through dedicated channels or through symporters as a byproduct of nutrient absorption. ENaC is the most prominent apical Na^+^ channel in the colon and allows for Na^+^ flux into the cell along its electrochemical gradient [5,38]. Comparable to the collecting duct of the kidney, its expression is regulated by aldosterone in response to hypovolemia [39]. Interestingly, aldosterone can also induce expression of ENaC in the small intestine [40,41]. Its modulation by CFTR has been subject of controversial discussion in the past [42,43,44,45]. It has been suggested that an increase in cAMP concentrations that in turn activates CFTR has an inhibitory effect on ENaC conductance [42,43,45]. However, the expression patterns of both channels in the mucosa do not support this hypothesis, as ENaC expression predominates in the colonic surface cells, whereas CFTR is mainly expressed in the crypt region [5]. Although this persistent controversy still awaits final clarification, CFTR seems to emerge as a potent regulator in nearly all pathways of electrolyte absorption discussed so far. 

The electrochemical gradient that confers Na^+^ absorption through ENaC is utilized by various symporters in the small intestine for secondary active nutrient transport into the enterocyte. Similar to its regulation in the kidney, glucose uptake in the proximal small intestine occurs through a member of the SGLT symporter family. The stoichiometry of the intestinal isoform (SGLT1) differs from the predominant renal isoform (SGLT2), as two Na^+^ ions are needed to carry one glucose molecule across the apical membrane [46,47,48]. The WHO issued oral rehydration salts (ORS) solution, which is used as a treatment for diarrheal diseases throughout the world, facilitates electrogenic Na^+^ uptake through SGLT1 by containing a particularly high concentration of glucose [47,49]. As water follows the absorbed electrolytes osmotically, this therapy has proven itself an effective strategy for rehydration. 

Currently, two inherited diseases that are associated with SGLT malfunction are known [47]. Mutations in SGLT1 are responsible for glucose/galactose malabsorption (GGM, OMIM 182380) resulting in osmotic diarrhea [50]. The second disorder, *i.e.*, familial renal glucosuria, affects SGLT2 mediated glucose transport in the kidney [51]. 

Amino acid transporters in the small intestine take advantage of the same Na^+^ gradient across the epithelia to conduct absorption. For a detailed review on the wide variety of involved transporters, please refer to a recent review [52].

### 2.2. Enterocyte Electrolyte Secretion

An estimated eight liters of fluid are being secreted into the intestine on a daily basis [2]. Although this fluid is also composed of gastric, pancreatic, and biliary juices as well as saliva, a significant portion of the secreted volume is considered to be *bona fide* enterocyte secretion [2]. Because it is not possible for enterocytes to actively transport fluid, secretion is linked to the creation of an osmotic gradient that drags water into the intestinal lumen. Apical chloride channels, such as CFTR, are the main generators of this osmotic driving force [2]. Chloride secretion through apical channels relies on a constant negative membrane potential that is generated by leak current through basolateral potassium channels and a steady intracellular concentration of chloride that is maintained by the basolateral Na^+^, K^+^, 2Cl^−^ (NKCC) symporter [2]. Although these basolateral proteins are crucial for the secretory function of the apical machinery, their review is omitted in the interest of conciseness. For an excellent review on chloride secretion, please refer to [2].

CFTR is a cAMP regulated chloride channel that is predominantly expressed in the intestinal crypt and to a lesser extent in the villus region [53,54]. The channel consists of two membrane spanning domains (MSDs), two nucleotide binding domains (NBDs) allowing for hydrolysis of ATP and a regulatory (R) domain that mediates regulatory interactions with PKA [55]. Regulation of CFTR mainly occurs through cAMP-dependent PKA at the R domain, but was also shown to be cGMP, PKC, c-src and calmodulin dependent [56,57,58,59,60,61,62]. In addition to on-site regulation, trafficking rates to the apical membrane are affected by cAMP, hence amplifying the impact of cAMP stimulation [63,64,65,66]. Recent work suggests that the R domain is pivotal for regulatory trafficking to occur [64].

Mutations in the CFTR gene result in manifestation of cystic fibrosis (CF), which represents the most prevalent genetic disorder in Caucasians. Although the majority of CF symptoms are related to respiratory pathologies, gastrointestinal manifestations of the disease are also frequent [67]. Meconium ileus, a postnatal intestinal obstruction resulting from hyperviscous stool, is often the first clinical sign of CF. The clinical findings and the correlating observations in knock-out animals that succumb to intestinal obstruction if not medicated with laxatives emphasize the major role of CFTR in the process of intestinal chloride/fluid secretion [67,68]. cAMP dependent chloride secretion has been demonstrated throughout the intestine using various experimental approaches and has been validated by its absence in CFTR mutants [2,3,69,70,71]. 

Ca^2+^ activated chloride channels (CaCCs) are extensively described as an alternate chloride efflux mechanism in colonic T84 cells [5,72,73,74,75]. The role of CaCCs in native human intestinal tissue is, however, more ambiguous as several groups reported a failure of intracellular Ca^2+^ to increase chloride secretion in CF patients [76,77,78]. Conversely, it has been suggested that a compensatory up regulation of CaCCs increases survival in CFTR mutant mice by alleviating symptoms of constipation [79]. The molecular identity of CaCCs has also been subject of controversial speculation. hCLCA1 had emerged as a possible candidate, as its expression had been demonstrated to be exclusively confined to the crypt, but was later shown to be a secretory protein rather than a channel [80]. Today, the list of candidates for CaCCs consists of TMEM16a, bestrophin-2, and hTTYH3 [81,82,83,84,85,86]. Functional evidence for their importance in intestinal chloride secretion has so far only been gained for TMEM16a in a knock-out mouse model [87]. Yet supplemental studies that define the significance of CaCCs in the human intestine remain to be conducted. 

ClC-2 channels may represent a third route for apical chloride secretion. CF mice with a mild intestinal phenotype showed residual apical chloride conductance, which was attributed to expression of ClC-2 [88,89]. However, additional disruption of ClC-2 in a ClC-2/CFTR double knock-out mouse failed to exacerbate intestinal symptoms in the affected animals [90]. Localization studies in human tissue further showed a supra-nuclear localization pattern that does not correlate well with the postulated secretory function of the channel [91]. Despite the ambiguous evidence for the involvement of ClC-2 in intestinal ion handling, the purportedly specific ClC-2 agonist lubiprostone is in clinical use to ameliorate symptoms of constipation. A recent study, however, demonstrates that intact CFTR is necessary for lubiprostone to exert its effect, further challenging the suggested importance of ClC-2 ion handling in the enterocyte [92]. 

## 3. The Pathophysiology of Enterotoxin Mediated Diarrhea

### 3.1. Enterotoxigenic *E. coli*

Enterotoxigenic *E. coli* (ETEC) infection is one of the leading causes of diarrhea in the developing world. Children are especially susceptible to intestinal infection, with 280 million cases of ETEC related diarrhea occurring each year in children under the age of five [93]. ETEC infection also accounts for 50–60% of all cases of traveler’s diarrhea, which amounts to about 60 million cases per year in total [94,95,96]. In light of these high incidence rates it comes as no surprise that ETEC infection takes its toll with an estimated 370,000 deaths each year, the vast majority of which are children [93]. 

ETEC produces at least one of two defined enterotoxins: heat stable enterotoxins (ST) or heat labile enterotoxins (LT). LT exists in two classes, only one of which is associated with intestinal disease, namely LT-I. LT-I is a hetero-oligomeric holotoxin, composed of one A subunit and five identical B subunits and thus belongs to the family of AB_5_ enterotoxins [97]. Among other bacterial toxins, the AB_5_ enterotoxin family also includes cholera toxin (CTX) and Shiga toxin (STX). Because these toxins exhibit a significant degree of homology in both sequence (the A1 subunits of CTX and LT-I have 78% sequence homology) and pathophysiology, we will regard LT-I and CTX as one entity with respect to their cellular mechanism of action [98]. The crystal structures of both toxins are now available to us and may aid in further elucidating the sites of action [99,100,101]. It should be noted that disease severity in ETEC infection is generally lower than in cholera infection. While the basic mechanisms for this effect are not fully understood, it has been proposed that the efficiency of toxin secretion may differ between the two bacterial species [102]. For the pathophysiological processes underlying LT-I infection, please refer to the section on *V. cholerae*.

ETEC produce ST enterotoxins in two variations: STa and STb. Consisting of only 18 or 19 amino acids, STs are considerably smaller in size than LTs and are monomeric in structure [103,104,105,106,107]. The STs contain six cysteine residues, forming three disulfide bonds [103,104,106,108,109]. The residues and bonds are responsible for the heat stability of the protein and were shown to be essential for its toxicity [108,109,110]. The structure of STs is shared to a significant degree with the heat stable toxins from *V. cholerae* O1/non-O1 and *Yersinia enterocolitica* [111,112,113]. Unlike LT-I, STs do not exert their toxic effects in the cytoplasm of the enterocyte, but rather bind to a receptor on the membrane surface. Receptor quantity decreases with age, which may account for the high prevalence and disease severity of ETEC mediated diarrhea in children [114]. The receptor has subsequently been cloned and identified as the membrane-bound guanylyl cyclase type C (GC-C), which is comprised of an extracellular receptor domain, a single membrane spanning domain, and an intracellular kinase homology domain followed by the guanylyl cyclase domain [115,116]. Stimulation of GC-C leads to an increase in intracellular cGMP concentrations, resulting in activation of PKA or cGMP-dependent protein kinase II (cGK II) and increased chloride secretion through apical CFTR channels [56,117,118]. Both kinases mediate CFTR activation and concomitant NHE3 inhibition via NHERF, leading to increased chloride secretion and reduced sodium absorption [17,19,56,119,120]. cGK (−/−) animals were further shown to secrete less fluid in response to STa exposure [118]. 

Diarrhea ensues as a product of increased luminal osmolarity. Apart from kinase mediated activation, cGMP may influence channel gating directly by binding to CFTR [121]. As CFTR is the common endpoint for both CTX and STa mediated diarrhea, CF patients and transgenic CF mice seem to have an innate immunity against both toxins [122,123]. The exact binding site of STa to GC-C is well characterized by the use of receptor mutagenesis and photoaffinity labeled STa [124,125]. Mutation analysis of STa has further revealed that the central β-turn region of the protein (Asn^11^–Cys^14^) is responsible for GC-C binding and that this interaction is dependent on hydrophobic amino acid sidechains [126,127,128,129]. A GC-C (−/−) model has helped provide us with compelling evidence of the cyclases’s involvement in the process of STa mediated diarrhea, as the affected animals were shown to be resistant against the effects of the toxin [130,131]. 

Beside STa, the endogenous peptides uroguanylin and guanylin, which are implicated in the maintenance of salt balance, serve as physiological activators of GC-C [132,133,134,135,136]. Guanylin is significantly homologous to STa in its structure and also relies on disulfide linkages for receptor activation [137]. Membrane guanylyl cyclases also serve as peptide hormone receptors regulating electrolyte homeostasis in other tissues. Atrio-natrioretic peptide, which is secreted by the atrium in response to increased atrial stretch, binds to a similar membrane guanylyl cyclase in the kidney [138]. GC-C has recently been identified as a pharmacological target in the treatment of constipation. The synthetic 14-peptide GC-C agonist linaclotide (formerly MD-1100) effectively alleviated symptoms of constipation in numerous clinical trials [139,140].

In addition to GC-C, several other STa receptors have been postulated to exist [141,142,143,144]. This hypothesis has recently been supported by the observation that STa can induce HCO_3_^-^ secretion in a non-cGMP, non-GC-C and non-CFTR dependent manner [145,146]. The fact that this HCO_3_^-^ secretion is not dependent on CFTR is especially remarkable, as intact CFTR is traditionally regarded to be a prerequisite for HCO_3_^-^ secretion [147]. 

Currently, two pharmacological strategies have been employed to specifically counteract the effects of STa: (i) inhibition of GC-C and its signaling; and (ii) inhibition of chloride secretion through CFTR [148,149,150,151,152]. The group of pyridopyrimidine derivatives has been demonstrated to decrease cGMP levels in T84 cells and to decrease STa triggered fluid secretion in an isolated intestinal loop model by inhibition of GC-C [148,149]. Specificity of the substance is limited, as other GC family members (GC-A,B and soluble forms) were also inhibited [148]. In addition, the tyrphostin tyrosin kinase inhibitors and the membrane permeable nucleoside analog 2-chloroadenosine were shown to inhibit GC-C activity in a cultured cell model [150,151,152]. However, the most promising substances for inhibiting STa mediated diarrhea are clearly the specific CFTR channel inhibitors, as they are non-absorbable and exert their effects on-site in the intestinal lumen. Except for one study, the majority of data has been generated in a CTX model, as CFTR represents the common endpoint for both CTX and STa mediated diarrhea [153]. Apart from specific treatment approaches, dietary calcium intake has been show to inhibit ETEC mediated diarrhea in human test subjects [154]. 

**Figure 1:**
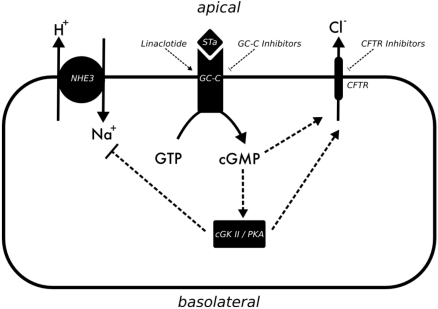
Model summarizing cellular processes during STa mediated diarrhea.

### 3.2. *Vibrio Cholerae*

In 2008, over 190,000 cases of cholera were reported to the WHO [155]. It is safe to say that the actual number of annual symptomatic *V. cholerae* infections is much higher as a result of underreporting. Particularly disconcerting is the fact that on average the number of cholera cases has been on the rise in recent years [155]. As the disease is transmitted feco-orally, cholera is associated with poor sanitary conditions. Cholera outbreaks are thus grim companions of natural catastrophes, political instability and poverty. The disease itself is characterized by a watery diarrhea with typical rice-water stools. The fulminant onset of diarrhea is extremely abrupt and the massive fluid loss of up to 1liter/h can be lethal within hours as a result of dehydration [156]. Although aggressive rehydration therapy has proven to be an effective countermeasure, the acute nature of the disease calls for rapid accessibility to treatment, which is often a challenging endeavor in underdeveloped regions. 

The effects of cholera are mediated by CTX, which is secreted by the bacterium. Once secreted, the B subunits of CTX bind to the membrane associated ganglioside GM1, which serves as a receptor for CTX on the cell surface. The number of B subunits correlates with toxicity, presumably by facilitating uptake through clustering of multiple GM1s [157]. Following binding, the CTX-GM1 complex undergoes endocytosis and embarks on a true odyssey through the enterocyte before the toxin finally exerts its pathological effects in the cytosol. The trafficking of CTX through the cell has received an extensive amount of scientific attention, with the hope of identifying a possible pharmacological target for the treatment of LT-I and CTX related diarrhea. The trafficking process is reviewed in great detail elsewhere and will only be described briefly in this review [158,159]. After endocytosis, CTX is trafficked via endosomes to the trans-Golgi network in an actin dependent manner [158,159,160]. The toxin subsequently continues its journey to the ER, where protein disulfide isomerase (PDI) unfolds the enzymatically active A1 chain of the holotoxin, allowing A1 to be released from the ER into the cytosol. The pathway through which A1 release occurs is not yet fully understood [158]. Once in the cytosol, A1 starts to exert its toxic effects. One of the earliest observations on the impact of CTX was made in Ussing chamber experiments on isolated rabbit ileal mucosa, demonstrating an inhibition of short circuit current (SCC; an indicator for chloride secretion) following toxin exposure [161]. Similar changes could be elicited by cAMP, suggesting that CTX exerts its action through elevation of intracellular cAMP concentrations [161]. The A1 chain elevates intracellular cAMP concentrations by activating the alpha subunit of a G-Protein (G_s_) which in turn increases adenylyl cyclase activity [102,162]. G_s__α_ loses its intrinsic GTPase activity by A1 mediated ADP ribosylation of an arginine residue, resulting in prolonged activity of G_s__α_ [163,164,165]. Cells from mice lacking the enzyme which antagonizes ADP ribosylation (ADP-ribosyl protein hydrolase) are verifiably more sensitive to CTX [166]. As outlined above, cAMP is an activator of apical CFTR channels, which was postulated to account for the observed changes in potential difference and SCC [102]. CTX triggered elevations of cAMP levels further inhibit Na^+^ uptake by NHE in the villus cell and thereby increase the osmotic driving force for fluid secretion [167,168]. 

Serotonin (5-HT) release from enterochromaffin cells has also been implicated in the mechanism of action of CTX [169,170,171]. A CTX mediated increase of 5-HT release was observed in a preparation of human ileum [169]. Application of the 5-HT_3_ receptor antagonist granisetron resulted in a decrease of both 5-HT release and fluid secretion in isolated rat intestinal segments [172]. In addition, CTX is capable of modulating the immune response of the host by eliciting IL-6 secretion from the enterocytes, depleting the reserves of CD8+ intraepithelial lymphocytes and by inhibiting CD4+ and CD8+ lymphocytes [173,174,175,176]. A recent study has also investigated the gene expression patterns in response to CTX exposure by micro-array analysis [177]. The affected genes are speculated to play a role in the innate defense mechanism of the exposed cell [177]. 

Prostaglandins of the E family (PGE) were shown to play an important role in the action of CTX mediated diarrhea. An increase of PGE synthesis was observed in rat and pig ileum in response to CTX exposure [178,179,180]. PGE is also able to induce fluid secretion in unexposed tissue [178,179] (this view has also been opposed by Hudson *et al.* [181]). The role of PGE in the genesis of cholera is further emphasized by the successful inhibition of secretion following treatment with cyclooxygenase (COX) inhibitors [182,183]. Cells from COX (−/−) animals, however, demonstrated no significantly different behavior from wild-type cells in terms of pathological fluid secretion [182]. To the present day, the exact relationship and hierarchy between the cAMP, PGE and 5-HT signals have not been thoroughly defined [102,184].

Although the mechanisms of intestinal electrolyte secretion are fairly well understood, the exact route of the ensuing water movement is still unknown [2]. Aquaporin water channels (AQP) have emerged as possible candidates for transcellular water movement [2]. Several groups have set out to investigate their role in cholera. CTX was shown to modulate permeability of several AQPs in an oocyte overexpression system [185]. AQP8 in particular is expressed in the intestine and was shown to be regulated by cAMP in the liver [186,187]. Furthermore, observations in rat intestine verify down regulation of AQP8 following CTX exposure [188](conversely, AQP10 is down regulated in human cholera patients [189]). However, results in a recent AQP8 (−/−) model do not support the hypothesis that AQP8 serves as an important route for water secretion in cholera, as the affected mice showed a normal secretory response to CTX [188].

Chloride secretion through CFTR is canonically regarded to be a major endpoint of CTX toxicity, presumably via elevation of intracellular levels of cAMP [102]. An early observation emphasizing this theory was made in CF mice that secreted less intestinal fluid in response to CTX [190]. Subsequently, it has been argued that the high prevalence of CF in our population may be a result of an innate resistance to cholera, thereby conferring an evolutionary advantage on heterozygous mutation carriers [190]. CFTR has evolved into a popular target for the potential treatment of cholera. Currently, three groups of specific CFTR inhibitors exist. The thiazolidinone CFTRInh-172 was shown to reduce CTX stimulated fluid secretion, assessed by intestinal weight measurements, by as much as 90% [153,191]. *In vivo* administration of the herbal extract SP-303 also yielded significantly less fluid accumulation, employing a comparable experimental approach [192]. The formulation of the original substance has recently been improved; however, *in vivo* data are not yet available [193]. The glycine hydrazide GlyH-101 was also tested in a closed loop model and reduced CTX induced fluid secretion by 80% [194]. Nonetheless, none of these substances is currently in clinical use. In the absence of a specific treatment for cholera, aggressive rehydration with ORS solutions remains the most crucial countermeasure against the massive fluid loss that occurs in the course of the disease. The efficacy of ORS treatment is exemplified in a case study from the 70s in which cholera mortality could be decreased among Bangladesh refugees to 3.6% by administration of ORS, compared to 30% in similar refugee camps that relied purely on intravenous fluid substitution therapy [195]. In addition to rehydration therapy, a single-dose administration of azithromycin has been shown to ameliorate disease severity [196,197]. Apart from these strategies, our specific therapeutic resources are limited. A supplemental pharmacological approach is still desirable. The specific CFTR inhibitors may represent a remedy for this need, but their clinical potency still remains to be reviewed.

**Figure 2:**
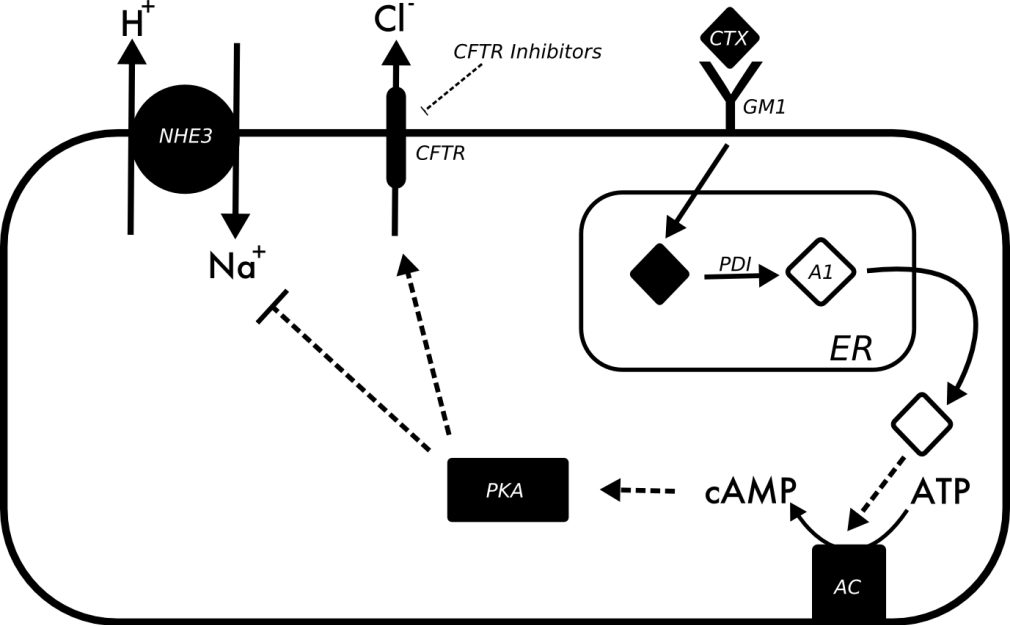
Model summarizing cellular processes during CTX mediated diarrhea.

### 3.3. Rotavirus

Rotavirus infection is the leading cause of diarrhea related hospitalization in children (<5 years)[198,199]. Estimations assert that 611,000 childhood deaths are caused annually by rotavirus, 80% of which occur in developing countries [200]. Rotavirus infection is a global phenomenon, and the industrialized world is not exempt from its burden. In the US alone, 60,000 hospitalizations per year are attributable to the virus, although mortality is substantially lower with 37 deaths annually [201].

The RNA of rotavirus encodes six viral proteins (VPs) and six nonstructural proteins (NSPs). One of the NSPs, namely NSP4, is a viral protein that induces diarrhea [202,203]. Although several other mechanisms, such as activation of the enteric nervous system, secretion of chemokines and epithelial ischemia, contribute to the diarrheagenic effects of rotavirus, we will limit our scope on the enterotoxin NSP4 [204,205,206]. NSP4 is a multifunctional protein and, besides its role as an enterotoxin, also serves as a receptor on the ER that mediates translocation of viral particles between cytosol and ER [207,208,209]. Recent data suggest that NSP4 can be trafficked from the ER to the plasma membrane where it is secreted by the infected enterocyte [210,211,212,213]. Given the fairly recent discovery of NSP4, our knowledge about its enterotoxic nature is still limited. We know that NSP4 can directly induce diarrhea and that this effect is not mediated through CFTR, as (−/−) animals were still susceptible to its effects [203,214]. In addition, it has been extensively described that NSP4 can increase intracellular Ca^2+^ concentrations [202,215,216]. It has recently been speculated that upregulation of the MS4A2 gene, which encodes a protein with a calcium channel activity, could play a role in this process [217]. Elevations of intracellular Ca^2+^ can activate CaCCs and may explain the vulnerability of CFTR (−/−) animals to NSP4. However, evidence for direct NSP4 induced intestinal chloride secretion is still scarce. So far, Ussing chamber studies in a cell culture model demonstrated an NSP4 dependent increase in SCC and I^-^ imaging revealed that NSP4 causes non-CFTR mediated anion permeability in isolated murine crypts [214,218]. 

A direct inhibition of SGLT1 has also been observed and may cause osmotic diarrhea by impaired uptake of Na^+^ and glucose, rather than by increased secretion [219]. A different group has reported that NSP4 can increase paracellular permeability in a polarized cell model [220]. In summary, conclusive information on the action of NSP4 on enterocyte ion transport is not available, although changes in Ca^2+^ homeostasis seem to be involved. 

As a result of the plethora of cellular mechanisms that are affected by Rotavirus infection, no specific treatment model exists. Yet, remarkable progress has been made in the prevention of the disease with the introduction of Rotavirus vaccines [221]. 

**Figure 3:**
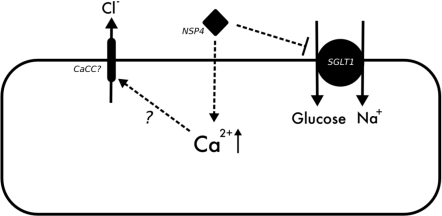
Model summarizing cellular processes during NSP4 mediated diarrhea.

## 4. Conclusions

Our knowledge of the molecular mechanisms underlying ETEC, cholera, and Rotavirus mediated diarrhea has massively improved over the last years. Paradoxically, our arsenal of potential specific therapeutic approaches is still small by comparison. The non-absorbable CFTR inhibitors can be regarded as the most promising advance in the direction of targeted treatment, but proof of their clinical efficacy and benefit is not yet available. Although unspecific, the advent of ORS represents the most revolutionary progress that has been achieved in terms of averting the lethal consequences of ADI in the past decades. The millions of remaining ADI related deaths, however, are a sad reminder that complimentary therapeutic approaches need to be developed and that accessibility to treatment has to be optimized. 
